# Health Care Utilization in Patients With Atopic Dermatitis Experiencing Topical Steroid Withdrawal: Observational Cross-Sectional Social Media Questionnaire Study

**DOI:** 10.2196/85183

**Published:** 2025-12-31

**Authors:** Alexander Shayesteh, Maja af Klinteberg, Sophie Vrang, Gunnthorunn Sigurdardottir, MariHelen Sandström Falk, Mikael Alsterholm

**Affiliations:** 1 Department of Public Health and Clinical Medicine, Dermatology and Venereology, Umeå University, Umeå, Sweden; 2 Swedish Asthma and Allergy Association, Stockholm, Sweden; 3 Department of Dermatology and Venereology in Östergötland, Linköping University, Linköping, Sweden; 4 Department of Biomedical and Clinical Sciences, Linköping University, Linköping, Östergötland, Sweden; 5 Vasakliniken Dermatology Clinic, Gothenburg, Sweden; 6 Department of Dermatology and Venereology, Sahlgrenska Academy, Institute of Clinical Sciences, University of Gothenburg, Gröna stråket 16, Gothenburg, 41345, Sweden, 46 31-342-1000

**Keywords:** atopic dermatitis, red skin syndrome, topical steroid addiction, topical steroid withdrawal, topical steroid withdrawal syndrome

## Abstract

**Background:**

Topical steroid withdrawal (TSW) is a controversial skin condition among health care providers due to a lack of evidence, but it has an impactful and growing presence on social media. There are few previous reports of health care utilization for symptoms attributed to TSW.

**Objective:**

This study aims to investigate health care utilization and requests as well as information sources for TSW among patients with atopic dermatitis (AD).

**Methods:**

This observational cross-sectional study used a questionnaire aimed at adults with AD, experiencing symptoms they attribute to TSW. The questionnaire was posted as a link, free to share with others, in a Swedish TSW−themed Facebook group and remained accessible for 4 weeks. Descriptive statistics and topical text analysis on open-ended items were used to present and interpret the results.

**Results:**

The participants (n=82) reported dermatologists (n=41, 50%), general practitioners (n=40, 49%), and practitioners of complementary and alternative medicine (CAM; n=32, 39%) as the most frequent health care contacts for TSW. However, among participants with ongoing symptoms attributed to TSW (n=68), ongoing health care contacts with general practitioners, dermatologists, and practitioners of CAM were reported by only 10% (n=7), 22% (n=15), and 13% (n=11), respectively. For symptoms attributed to AD, the frequencies of health care provider contacts were higher. Almost all participants had sought help from a general practitioner (n=81, 99%) or a dermatologist (n=76, 93%) at some point, and many had also consulted a practitioner of CAM (n=59, 72%). Among those with ongoing symptoms attributed to AD, 43% (n=26) had an ongoing contact with a dermatologist. Participant-requested help and support from health care providers included understanding and confirmation of TSW impairments (n=45, 56%), treatment of symptoms (n=26, 32%), and increased awareness and information about TSW from health care providers (n=21, 26%). The most common TSW information sources were Facebook (n=78, 96%), websites (n=75, 93%), and Instagram (n=45, 56%), but YouTube (n=11, 14%), podcasts (n=7, 10%), and TikTok (n=5, 6%) were also reported.

**Conclusions:**

This study investigates health care utilization patterns related to TSW. The results indicate that the participants received insufficient support from health care providers for symptoms they attributed to TSW. The participants initiated and maintained health care provider contacts for symptoms attributed to AD to a greater extent than for TSW and sought information and support for TSW elsewhere. Targeted interventions to overcome this could be educational efforts for general practitioners and dermatologists about the current scientific knowledge of TSW as well as the TSW discourse on social media. In addition, health care providers need to engage and contribute to evidence-based content about TSW on relevant social media platforms to prevent the spread of misinformation about topical glucocorticoids.

## Introduction

### Background

Topical steroid withdrawal (TSW) is described as an adverse skin reaction following the tapering or discontinuation of potent topical glucocorticoids (TGCs) [1,2]. It has also been reported to occur during TGC use [3]. The skin becomes intensely red, with itching, stinging, or pain [4,5]. Large skin areas are affected with frequent involvement of the face, and specific signs such as “red sleeve” (involvement of the arms with sparing of the hands) and “elephant wrinkles” (thickened skin with reduced elasticity over joints) have been documented [6]. Although first described in the late 1960s, TSW is a concept that has attracted rapidly increasing attention and concern among patients in the era of social media [3,7-9]. Meanwhile, TSW remains controversial among health care providers as there is neither clear evidence to support the suggested mechanism of addiction followed by symptoms of withdrawal nor a clinical definition of the condition [7,10-12]. A suggestion for the management of symptoms attributed to TSW, including cessation of TGCs, psychosocial support, and systemic treatment with dupilumab, was just provided using the modified Delphi consensus method [13]. Recently, steroid-induced dysregulation in nicotinamide adenine nucleotide metabolism has been demonstrated in clinical samples and proposed as an explanation for the symptoms described in TSW [14]. The conclusions drawn from this small study have been criticized [15].

Some aspects of health care utilization for symptoms attributed to TSW have been described. In retrospective reviews of case notes for patients in the United Kingdom and Australia, complementary medical practices, psychology support, and online research were reported [5,16]. Qualitative studies have explored health-seeking behavior and interactions with medical professionals and found patient withdrawal from standard dermatology care and experiences of dismissal when voicing TSW concerns [17,18]. Social media is abundant with TSW content, and it is assumed that this serves as a major source of information and support for patients [9,19,20]. The TSW discourse on social media is mostly based on accounts from individuals who identify as sufferers of TSW, with little or no contribution from health care providers [8,9,21,22].

### Prior Work

The lack of a definition makes the TSW population hard to identify and reach within the health care system. Therefore, we conducted a questionnaire study on Swedish social media aimed at individuals with atopic dermatitis (AD) who had experienced symptoms that they attributed to TSW. We have previously reported that the investigated cohort was young, predominantly female, mostly self-diagnosed with TSW, and that the skin signs and symptoms that they attributed to TSW caused a significant negative life impact [4].

### Study Objectives

The aim of this study was to describe TSW-related health care utilization, requests, and information sources as reported by our previously characterized cohort. The results could help to define targets for intervention to improve the management of TSW.

## Methods

### Questionnaire Design and Distribution

A 47-item questionnaire was constructed in Swedish in SurveyMonkey (SurveyMonkey Inc), as described elsewhere [4]. Checklist 1 describes the study design and reporting with the CHERRIES (Checklist for Reporting Results of Internet E-Surveys) [23]. In short, the questionnaire was designed and modified in a stepwise fashion where items were consecutively evaluated by the authors, a focus group of dermatologists from the steering group of SwedAD (the Swedish nationwide registry for patients with AD receiving systemic pharmacotherapy), and a focus group of patients with AD recruited from the Swedish Asthma and Allergy Association. Questionnaire items were multiple-choice or open-ended. Multimedia Appendix 1 features the informed consent and the questionnaire, translated into English. Adaptive questioning was used. Figure S1 in Multimedia Appendix 2 shows the flowchart for questionnaire items.

It was not considered feasible or appropriate to recruit patients in a clinical setting as there are no acknowledged diagnostic criteria for TSW. In addition, it has been reported that individuals with symptoms they attribute to TSW can feel dismissed in health care settings [18]. Clinical recruitment could therefore have excluded a substantial portion of the target population or only captured those already engaged in dermatological care. To reach individuals with relevant experiences, the questionnaire was instead fielded in a Swedish private TSW−themed Facebook group. At the time, this was the largest Swedish online community for TSW known to the authors. The administrator of the Facebook group was contacted by the patient representative in the research group (SV) with a request to post a link to the questionnaire [24]. The post with the link was made on April 24, 2023, describing the aim of the study, that individuals aged 18 years or older with previous or ongoing AD combined with previous or ongoing symptoms attributed to TSW were eligible as respondents, and encouraging dissemination of the link through sharing on social media where relevant. Reposts were made on May 9, 2023, and May 17, 2023, as reminders. The questionnaire remained open for 4 consecutive weeks.

### Inclusion Criteria

The inclusion criteria were age ≥18 years, previous or ongoing symptoms attributed to AD, and previous or ongoing symptoms attributed to TSW. The questionnaire was terminated if an answer did not meet the inclusion criteria.

### Topical Text Analysis

A topical text analysis was performed on the answers to open-ended items as previously described [4,25]. Topical text analysis refers to the identification of recurring themes and keywords in qualitative responses. Briefly, AS and MaK independently identified keywords and topics, whereafter consensus was reached through discussion. The result was triangulated with the other authors to identify and mitigate biases and strengthen the interpretations.

### Data Analysis

IBM SPSS Statistics for Windows (version 28.0.1.1 [15]) was used for descriptive statistics. The results are shown as the number and percentage observed per category. Any missing data are indicated by presenting the total number observed for each item in tables and figures.

### Ethical Considerations

Ethical approval for the study, including the study protocol, was sought with a request for an advisory statement from the Swedish Ethical Review Authority. As the study did not involve any intervention or processing of identifiable personal data, it was not subject to formal ethical review under Swedish legislation [26]. The advisory statement from the Swedish Ethical Review Authority confirmed that there were no ethical objections to the study (application number Dnr 2023-00189-01).

When accessing the link to the questionnaire, the participants were presented with an informed consent statement that described the aim and method of the study, what was required from the participant, and the management of data. The informed consent statement translated from Swedish into English is available in Multimedia Appendix 1. The participants gave informed consent by proceeding to the questionnaire after taking part in the informed consent statement. No compensation was given to the participants. The participants were anonymous to the investigators and could not be identified or contacted.

## Results

The questionnaire was entered by 98 individuals and completed by 82, hereafter referred to as participants. Figure S2 in Multimedia Appendix 2 shows the flowchart for the completion rate.

**Figure 1.**
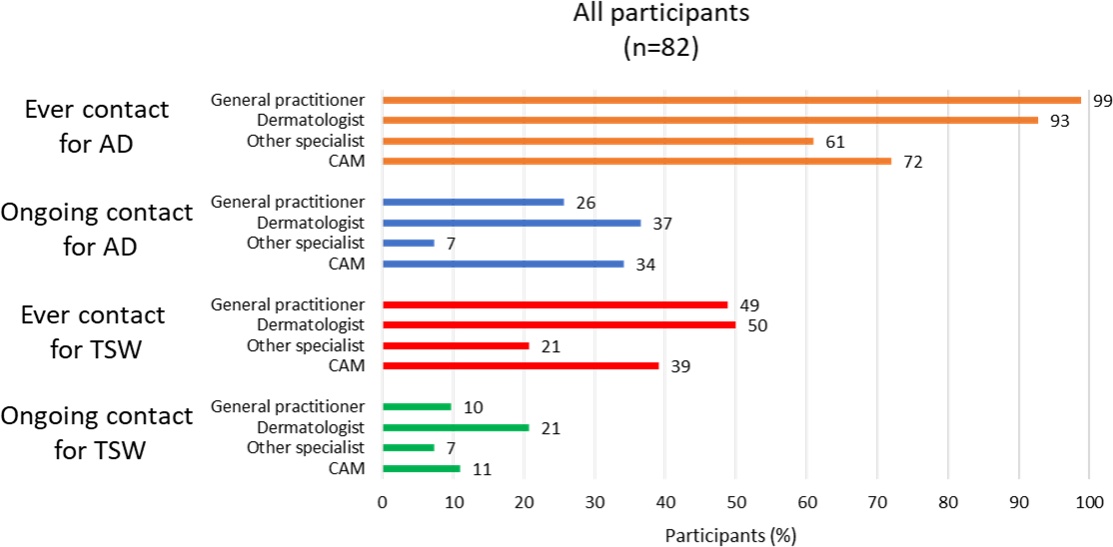
Frequencies of self-reported health care contacts for atopic dermatitis (AD) and topical steroid withdrawal (TSW). The data are from all participants in a Swedish observational, cross-sectional, social media questionnaire investigating TSW health care utilization, requests, and information sources in adults with AD combined with symptoms attributed to TSW. The link to the questionnaire was posted on April 24, May 9, and May 17, 2023, in a Swedish TSW−themed private Facebook group. Sharing of the link with others to reach as many individuals with relevant experiences as possible was encouraged in the posts. The questionnaire was open from April 24 to May 21, 2023. Participants could report multiple health care contacts. CAM: complementary and alternative medicine.

Figure 1 illustrates health care utilization for symptoms attributed to TSW and AD. The participants were asked to define any reported health care contact as “previous—not ongoing” or “ongoing.” The most frequently reported previous or ongoing health care contacts for symptoms attributed to TSW among all participants were dermatologists (n=41, 50%), general practitioners (n=40, 49%), and practitioners of complementary and alternative medicine (CAM; n=32, 39%).

**Figure 2.**
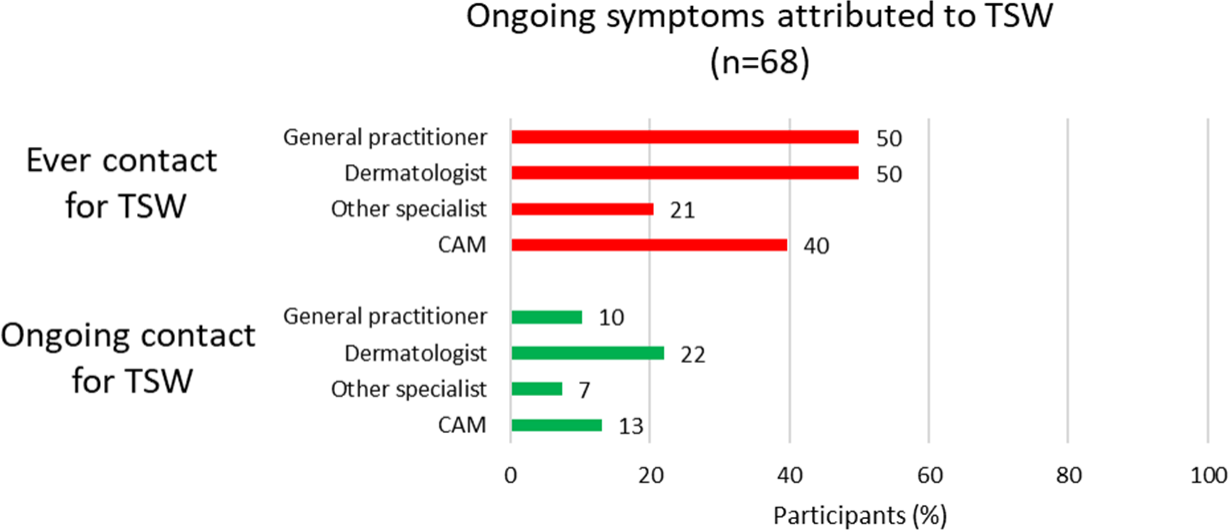
Frequencies of self-reported health care contacts for topical steroid withdrawal (TSW) among participants with ongoing symptoms attributed to TSW. The data are from a Swedish observational, cross-sectional, social media questionnaire investigating TSW health care utilization, requests, and information sources in adults with atopic dermatitis (AD) combined with symptoms attributed to TSW. The link to the questionnaire was posted on April 24, May 9, and May 17, 2023, in a Swedish TSW−themed private Facebook group. Sharing of the link with others to reach as many individuals with relevant experiences as possible was encouraged in the posts. The questionnaire was open from April 24 to May 24, 2023. As 53 participants reported both ongoing AD and TSW, the groups in Figures 2 and 3 overlap. Participants could report multiple health care contacts. CAM: complementary and alternative medicine.

Figure 2 shows that among participants with ongoing symptoms attributed to TSW (n=68), ongoing health care contacts with dermatologists, general practitioners, and practitioners of CAM were only reported by 22% (n=15), 10% (n=7), and 13% (n=11), respectively.

The participants could report none, any, or all of the item options “General practitioner,” “Dermatologist,” “Other specialist,” and “Practitioner of CAM.” Other specialists were reported as previous or ongoing contacts by 61% (n=50) and 21% (n=17) of participants for AD and TSW, respectively. This option could be specified in free text, but few participants chose to do so. For AD, pulmonologist or allergist (n=2), pediatrician or pediatric allergist (n=2), ophthalmologist (n=1), and nutritionist (n=1) were mentioned. For TSW, allergist (n=3) was mentioned.

No health care contact ever for symptoms attributed to TSW was reported by 46% (n=38). Despite ongoing symptoms attributed to TSW, 68% (46/68) reported no ongoing health care contact for those symptoms. All participants with any previous or ongoing health care contacts for symptoms attributed to TSW (54%, n=44) had consulted 2 or more of the categories “General practitioner,” “Dermatologist,” “Other specialist,” and “Practitioner of CAM.”

**Figure 3.**
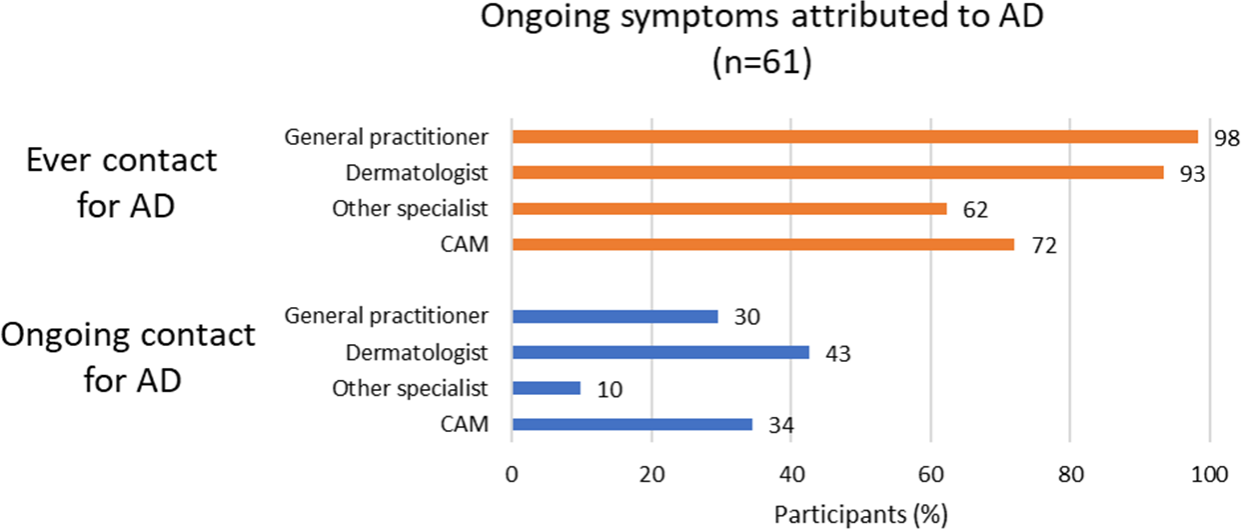
Frequencies of self-reported health care contacts for atopic dermatitis (AD) among participants with ongoing symptoms attributed to AD. The data are from a Swedish observational, cross-sectional, social media questionnaire investigating TSW health care utilization, requests, and information sources in adults with AD combined with symptoms attributed to TSW. The link to the questionnaire was posted on April 24, May 9, and May 17, 2023, in a Swedish TSW−themed private Facebook group. Sharing of the link with others to reach as many individuals with relevant experiences as possible was encouraged in the posts. The questionnaire was open from April 24 to May 21, 2023. As 53 participants reported both ongoing AD and topical steroid withdrawal, the groups in Figures 2 and 3 overlap. Participants could report multiple health care contacts. CAM: complementary and alternative medicine.

Table 1 describes investigations for symptoms attributed to TSW. Investigations beyond medical history and physical examination of the skin were unspecified blood tests (14/44, 32%), contact allergy tests (13/44, 30%, previously reported [4]), and skin biopsies 7% (3/44).

Figure 1 shows that both previous and ongoing health care contacts with general practitioners, dermatologists, other specialists, and practitioners of CAM were more common for symptoms attributed to AD than for symptoms attributed to TSW. Most participants had consulted a general practitioner (n=81, 99%) or a dermatologist (n=76, 93%) at some point, and many had also consulted a practitioner of CAM (n=59, 72%) or other specialists (n=50, 61%). Figure 3 illustrates that among participants with ongoing symptoms attributed to AD (n=61), ongoing health care contacts with dermatologists, general practitioners, and practitioners of CAM were reported by 43% (n=26), 30% (n=18), and 34% (n=21), respectively.

**Table 1.** Investigations for symptoms attributed to topical steroid withdrawal (TSW)^a^.

Investigations	Participants, n (%)
Medical history on previous diseases and treatments	19 (43)
Physical examination of the affected skin areas by a doctor	19 (43)
Physical examination of all skin areas by a doctor	10 (23)
Skin biopsy	3 (7)
Blood test	14 (32)
Contact allergy test (applied on your back and checked after a few days)	13 (30)
No investigations have been performed	13 (30)
Other actions (please specify)	12 (27)^b^

a The data are from a Swedish observational, cross-sectional, social media questionnaire investigating TSW health care utilization, requests, and information sources in adults with atopic dermatitis combined with symptoms attributed to TSW. The link to the questionnaire was posted on April 24, May 9, and May 17, 2023, in a Swedish TSW−themed private Facebook group. Sharing of the link with others to reach as many individuals with relevant experiences as possible was encouraged in the posts. The questionnaire was open from April 24 to May 21, 2023. The table shows the frequencies of answers to the multiple-choice item “What investigations have been performed by your health care provider in regard to your TSW?” for the 44 participants who reported contact with a health care provider for symptoms attributed to TSW.

b The free-text answers given under the option “Other actions” were general comments about the participants’ experiences and did not specify any investigations.

Table 2 describes the participants’ requests for help and support for symptoms attributed to TSW. This was investigated with topical text analysis of the answers to the item “What kind of help and support would you like from the health care system?”. The most mentioned topics were “Support, understanding, and confirmation of TSW impairments” (n=45, 56%), “Medical help to alleviate symptoms” (n=26, 32%), and “Increased awareness and information about TSW from health care providers” (n=21, 26%). “Medical investigation and a diagnosis” (n=8, 10%) and “Innovative treatments and research” (n=4, 5%) were less requested.

Table 3 presents the participants’ self-reported TSW information sources. Social media platforms (Facebook: n=78, 96%; Instagram: n=45, 56%), and TSW-themed websites (n=75, 93%) were the most reported sources. YouTube and TikTok were not included as options but were mentioned under “Other options” by 14% (n=11) and 6% (n=5), respectively.

**Table 2.** Requested help and support for symptoms attributed to topical steroid withdrawal (TSW)^a^.

Requested help and support	Participants, n (%)	Example of answers
Support, understanding, and confirmation of TSW impairments	45 (56)	“I need them (the healthcare providers) to understand and show empathy for my problems.”
Medical help to alleviate symptoms	26 (32)	“We need a treatment programme for TSW.”
Increased awareness and information about TSW from health care providers	21 (26)	“Someone educated in TSW should be on my side. As it stands now, I am told that TSW does not exist.”
Paid sick leave	11 (14)	“I need paid sick leave. TSW patients should be able to rest in a stress-free environment.”
Medical investigation and a diagnosis	8 (10)	“I want a proper medical investigation regarding causes, triggers, and cross-reactions in TSW.”
Innovative treatments and research	4 (5)	“We need support for unconventional treatments and more research since some are affected by TSW while others are not.”

a The data are from a Swedish observational, cross-sectional, social media questionnaire investigating TSW health care utilization, requests, and information sources in adults with atopic dermatitis combined with symptoms attributed to TSW. The link to the questionnaire was posted on April 24, May 9, and May 17, 2023, in a Swedish TSW−themed private Facebook group. Sharing of the link with others to reach as many individuals with relevant experiences as possible was encouraged in the posts. The questionnaire was open from April 24 to May 21, 2023. The table shows the topical text analysis of the answers to the item “What kind of help and support would you like from the health care system?”. The frequencies of mentioned topics are presented, along with examples of answers. Participants (n=81) could mention more than 1 topic.

**Table 3.** Self-reported topical steroid withdrawal (TSW) information sources^a^.

Information source	Participants, n (%)
Facebook groups	78 (96)
TSW-themed websites	75 (93)
Instagram	45 (56)
YouTube	11 (14)
Books or magazines or journals	11 (14)
Podcasts	8 (10)
TikTok	5 (6)
Blogs and online forums	4 (5)
TV or radio	N/A^b^
Twitter (now X)	N/A

a The data from a Swedish observational, cross-sectional, social media questionnaire investigating TSW health care utilization, requests, and information sources in adults with atopic dermatitis combined with symptoms attributed to TSW. The link to the questionnaire was posted on April 24, May 9, and May 17, 2023, in a Swedish TSW−themed private Facebook group. Sharing of the link with others to reach as many individuals with relevant experiences as possible was encouraged in the posts. The questionnaire was open from April 24 to May 21, 2023. The multiple-choice item “Which sources have you utilized for information about TSW?” was answered by 81 participants.

b N/A: not available.

## Discussion

### Principal Findings

This study investigates health care utilization for TSW, a primarily self-diagnosed condition where support and advice on management are offered by patient advocacy groups and users on social media platforms [27-29].

The frequencies of both previous and ongoing health care contacts were lower for symptoms attributed to TSW than for AD across all types of health care providers. Even with ongoing symptoms, it was less common to have a current health care contact for TSW than for AD. We have previously described that the participants reported prominent and widespread skin signs, accompanied by pruritus, pain, and sleep disturbance, causing a considerable negative impact on social and intimate relations, anxiety, and feelings of depression [4]. It is striking and unfortunate that ongoing health care contacts were rare in relation to these severe manifestations. Ongoing AD treatment with systemic pharmacotherapy (dupilumab, methotrexate, upadacitinib) was reported by 22% (n=15) and phototherapy by 15% (n=12) of the participants, as previously presented [4].

The participants were more likely to initiate and maintain health care contacts for symptoms that they attributed to AD than for symptoms that they attributed to TSW. Presumably, this was partly explained by unmet needs in the management of TSW. The participants requested validation of their symptoms and suffering, targeted medical treatment, and information about TSW from health care providers. In addition, we have previously reported that reasons for not seeking medical help for TSW in this cohort were fear that the medical staff would not recognize the existence of TSW and a lack of knowledge of the condition [4]. Low confidence in health care providers’ ability to offer the right treatment, experience or fear of being dismissed when expressing concerns regarding TGC safety, and being advised not to consult a health care provider were other reasons (data not shown).

The skin signs and symptoms attributed to TSW are similar to those described in insufficiently treated AD [30]. As the participants reported more ongoing health care contacts for AD than for TSW, even when they considered both conditions to be active, it can be speculated that some participants chose not to discuss the symptoms they attributed to TSW with their health care provider or that their concerns were ignored. TGCs are often part of the recommended treatment for AD. This can cause a dilemma for a patient with an unspoken attribution of symptoms to TSW, leading to nonadherence to treatment or ending of the health care contact.

For AD, ongoing contact with a practitioner of CAM and ongoing contact with a general practitioner were reported with a similar frequency, perhaps reflecting a tendency to seek health care with less focus on TGCs. On the other hand, ongoing contact with practitioners of CAM and ongoing contact with general practitioners were equally common for symptoms attributed to TSW as well, although at a lower level than for AD. Overall, 72% (n=59) had consulted a practitioner of CAM for AD and 39% (n=32) for symptoms attributed to TSW at some point. This is higher than what has been reported in an Australian cohort where only 6/55 (11%) had consulted a practitioner of CAM and, for the TSW-specific consultation, comparable to 9/19 (47%) reported from a clinic in the United Kingdom [5,16]. In Swedish surveys aimed at the general population, the use of any type of CAM, including self-prescribed and self-administered treatment, has been reported by 64% (318/500) to 71% (1089/1534) of the respondents [31,32]. Among those, 13% (65/500) to 33% (505/1534) reported consultation with a practitioner of CAM. The high levels of CAM consultations among the participants in this study should be interpreted in the context of the relatively common use of CAM services in Sweden but suggest that the participants were more inclined to consult with a practitioner of CAM than the general population. Variation in health care systems, cultural attitudes, and accessibility of CAM services could account for differences with comparable cohorts elsewhere.

Interestingly, only 10% (n=7) of the participants expressed a need for the investigation of the cause of their symptoms. It has been shown that patients tend to value and trust their own experiences of TGCs and may perceive health care providers’ emphasis on the safety of TGCs as dismissive [18]. We have previously reported that 93% (76/82) of the participants in this study regarded TGCs as the principal trigger factor for their symptoms, with 33% (n=27) also identifying oral glucocorticoids as triggering [4]. Investigating other explanations, including but not limited to those related to TGCs, poses a challenge for health care providers. Undergoing skin biopsies and patch tests to address concerns of TSW was reported by a minority of the participants. Investigations are needed to understand symptoms attributed to TSW and should be offered when relevant. Still, it is likely that some patients may decline investigation and choose self-management strategies.

The reported health care utilization pattern in this study suggests that symptoms attributed to TSW were something that the participants dealt with on their own to a greater extent than symptoms attributed to AD. With limited scientific literature and almost no TSW information available from health care providers, patients turn to social media and TSW-dedicated websites for information and support [3,19,27]. Facebook was the most frequently reported source of TSW information, which likely reflects that the questionnaire was posted in a TSW-themed Facebook group. TSW-themed websites and Instagram were the 2 other major sources of information. It can be assumed that the participants’ age (74% [61/82] were 18‐39 years old) and the time of data collection (spring 2023) influenced the outcome. For instance, TikTok would probably have been reported by a higher number of participants in 2025. TikTok now has more than 1 billion monthly users, and its potential for sharing public health dermatology information has been recognized [33-35]. The TSW content on TikTok, where #topicalsteroidwithdrawal has more than 600 million views, was recently assessed with instruments for quality-grading of health care−related digital content [21]. The authors found that the 100 most viewed TSW videos were of low quality, lacked pertinent information, and were based on personal accounts with no health care provider engagement.

### Strengths and Limitations

A strength of this study is that it was designed to reach as many individuals with relevant experiences as possible. Social media was chosen to field a free-to-share questionnaire because there are no established diagnostic criteria for TSW, making it hard to define and reach the intended population in the health care system [36,37]. The questionnaire was detailed, with a high completion rate, allowing the description of various aspects of TSW, some of which have been published [4]. For instance, the manifestations attributed to TSW and their life impact were investigated, which was helpful in the assessment of the relevance of the health care contacts reported here.

Limitations of the study design are that all data were self-reported and that a response rate cannot be calculated. Recall bias and confirmation bias can affect data in controversial topics such as TSW. The results must be interpreted with caution as the external validity is unknown. The demography and attitudes of the participants are likely to reflect that a dedicated Facebook group was chosen to launch the questionnaire.

Multiple-choice items were used to investigate some aspects of TSW, which can introduce bias or loss of information. For instance, the item option “Other specialist” was not specified by most participants, but this information can be important as TSW-related complications requiring ophthalmologists have been reported [38]. No participants reported having seen a psychologist; however, this has been reported as high as 22% in the literature and noted in case reports [20,38,39]. The reason for this difference is not clear, but possible explanations are questionnaire design and the Swedish health care system. In retrospect, it might have been better to include specific items about psychological and psychiatric support. On the other hand, given the sensitive nature of mental health and the challenges in accessing individuals with TSW, including such items could have increased participant discomfort, the risk of social desirability bias, or non-response. In the Swedish public health care system, a referral (usually from a general practitioner) is required for outpatient psychiatrist and psychologist consultations and can be unavailable or expensive in private practice.

A larger number of open-ended items could have provided more data but at the risk of a lower completion rate. No rating of the severity of AD was included, making it difficult to assess if the frequencies of ongoing health care contacts were reasonable given the symptoms.

### Conclusions

As research data on TSW begin to emerge, it is important for clinical researchers, health care providers, and patient advocacy groups to consider how and where the information should be put forward. As of now, patients navigate parallel realities. Social media is abundant with engaging and supportive TSW content of low quality, and sometimes even misinformation. Health care providers are unfamiliar with the TSW discourse and have difficulties in engaging in conversations about TSW. Interventions are needed to bridge this discrepancy [40]. For health care providers, it is a delicate task to increase engagement in the TSW discussion without inadvertently confirming theories that lack scientific support.

The pattern of health care contacts in this study suggests that education about the evidence base for TSW, including the social media discourse, should be aimed at general practitioners and dermatologists. Even though TSW is not confirmed as a distinct entity, health care providers must be equipped to discuss the topic and engage empathically with concerned patients.

The information sources reported here support that health care provider organizations should provide evidence-based information about TGCs and TSW on relevant social media platforms and other digital services to reach those with general concerns about TGCs and those who have symptoms that they attribute to TSW.

## Supplementary material

10.2196/85183 Multimedia Appendix 1 Informed consent and presentation of the questionnaire (translated from Swedish into English).

10.2196/85183 Multimedia Appendix 2 Flowchart for questionnaire items and flowchart for questionnaire participation.

10.2196/85183 Checklist 1 CHERRIES checklist.

## Abbreviations

AD: atopic dermatitis
CAM: complementary and alternative medicine
CHERRIES: Checklist for Reporting Results of Internet E-Surveys
TGC: topical glucocorticoid
TSW: topical steroid withdrawal
