# Supplementary material for: Health Care Utilization in Patients With Atopic Dermatitis Experiencing Topical Steroid Withdrawal: Observational Cross-Sectional Social Media Questionnaire Study
Source: JMIR Form Res. 2025 Dec 31;9:e85183. doi: 10.2196/85183 (PMC12755344; doi:10.2196/85183)
Supplement: Multimedia Appendix 1 [file formative-v9-e85183-s001.pdf]

Multimedia Appendix 1. Informed consent and presentation of the questionnaire (translated from Swedish into English).

A circle in front of item response options indicates that the participant could choose one option. A square in front of item response options indicates that the participant could choose one or more options. The questionnaire was automatically terminated if a participant chose any response option that did not met the inclusion criteria: previous or ongoing atopic dermatitis, and previous or ongoing topical steroid withdrawal.

Informed consent

Information regarding the research project 'Experiences of topical steroid withdrawal (cortisone addiction) among individuals with atopic dermatitis – a digital questionnaire'

We kindly ask if you would consider participation in the research project presented here.

What is the purpose of the project and why do you want me to participate?
The questionnaire is aimed at individuals with atopic dermatitis (atopic eczema) who have current and/or prior experience of suspected or confirmed topical steroid withdrawal (TSW). TSW can be described as an unforeseen skin reaction during or after treatment with cortisone cream. There is a lack of research-based knowledge regarding TSW. This is why your experience of TSW is important!

Our research group consists of doctors, nurses, and patient representatives who work in clinical dermatology, do research on atopic dermatitis, and educate medical staff. We are members of the steering group for SwedAD, the Swedish quality registry for atopic dermatitis.

We want to investigate the symptoms associated with TSW, how those symptoms affect your life, and prior and current treatments and healthcare contacts for atopic dermatitis and TSW.

The research principal is the Västra Götaland region. A research principal is the organisation responsible for the research project. The Swedish Ethical Review Authority has approved the project (Dnr 2023-00189-01).

How is the project performed?
Your participation is anonymous. We ask you to fill out a digital questionnaire on one occasion. This takes approximately 10–15 minutes. By filling out the questionnaire, partly or in its entirety, you consent to your answers being part of the data which will be analysed and presented.

What will happen to my answers?
The answers you give anonymously in the questionnaire are the only data collected. No other information will be gathered or used in the analysis and presentation. Your answers cannot be traced back to you by the research group or anyone else. Your answers are saved on a password-protected computer behind a firewall and are only accessible to those researchers who will analyse and present the data.

If you are not satisfied with the way in which your data are processed, you have the right to file a complaint with the supervisory authority, IMY, the Swedish Authority for Privacy Protection.

How do I receive information about the results of the project?

The research group aims to publish the results of the project in a peer-reviewed scientific journal. Unfortunately, it will not be possible for us to inform you personally of the publication, as your participation is anonymous.

Participation is voluntary

By going forward and answering this questionnaire, you have consented to us using your anonymous answers in the research project.

Head of the research group

Mikael Alsterholm, senior consultant
Department of Dermatology and Venereology
Sahlgrenska University Hospital
413 45 Gothenburg
[e-mail address/phone number]

Questionnaire items

1. I am…
   ○ Male
   ○ Female
   ○ I don’t identify as male or female

2. My age is…
   ○ 18–29 years
   ○ 30–39 years
   ○ 40–49 years
   ○ 50–59 years
   ○ 60 years or older

3. Which is your highest level of education?
   ○ Elementary school
   ○ High school
   ○ Other post-secondary education
   ○ University < 3 years
   ○ University ≥ 3 years
   ○ None of the above

4. Which of the following diseases are present or have been present in **your family**?
   □ Asthma
   □ Allergy symptoms from the nose and eyes
   □ Eczema due to contact allergy
   □ Hives
   □ Atopic dermatitis
   □ Unknown
   □ None of the above

5. Do you have, or have you previously had, atopic eczema?
   ○ Yes
   ○ No → QUESTIONNAIRE TERMINATED

6. Are you <u>currently</u> experiencing symptoms of atopic eczema?

   ○ Yes

   ○ No

7. Which of the following diseases do you have, or have you had?

   □ Asthma

   □ Allergy symptoms from the nose and eyes

   □ Eczema due to contact allergy

   □ Hives

   □ Other diseases (please specify) → FREE-TEXT ANSWER POSSIBLE

   □ I have not had any previous diseases

8. How old were you when the first symptoms of atopic eczema appeared?

   ○ Child (< 2 years)

   ○ Child (2–6 years)

   ○ Child (7–12 years)

   ○ Teen (13–17 years)

   ○ Adult (18 years or older)

   ○ Unknown

9. How were you diagnosed with atopic eczema?

   ○ By a general practitioner

   ○ By a dermatologist

   ○ By another specialist practitioner

   ○ By a nurse

   ○ By family or friends

   ○ I made the diagnosis myself

   ○ Unknown

In the following, you will find questions about your atopic eczema.

Which healthcare provider/s have you consulted for atopic eczema?

10. General practitioner

   ○ Previous contact, not ongoing

   ○ Ongoing contact

   ○ Never had contact

11. Dermatologist
    ○ Previous contact, not ongoing
    ○ Ongoing contact
    ○ Never had contact

12. Other specialist doctor (for example paediatrician, allergist)
    ○ Previous contact, not ongoing
    ○ Ongoing contact
    ○ Never had contact
    ○ If you have had or have contact with other specialist care, please describe what type → FREE-TEXT ANSWER

13. Practitioner of complementary medicine (used in addition to prescribed treatment) and/or alternative medicine (used instead of prescribed treatment), such as natural remedies, supplements, diets, acupuncture, homeopathy or other
    ○ Previous contact, not ongoing
    ○ Ongoing contact
    ○ Never had contact

In the following, you will find questions about your treatment for atopic eczema.

14. Do you have <u>ongoing</u> treatment for atopic eczema?
    ○ Yes
    ○ No (please explain why) → FREE-TEXT ANSWER + FORWARDED TO ITEM 20

15. If you have <u>ongoing</u> treatment for atopic eczema, which of the following are you using?
    □ Ointments/creams/lotions/pharmaceutical solutions
    □ Tablets
    □ Injections
    □ Medical UV therapy
    □ Tanning booth
    □ Other treatment

16. If you have <u>ongoing</u> treatment with ointments/creams/lotions/pharmaceutical solutions which of the following are you using?
    □ Emollients
    □ Calcineurin inhibitors (Protopic/tacrolimus or Elidel/pimecrolimus)
    □ Cortisone

□ I don’t use ointments/creams/lotions/pharmaceutical solutions

17. If you have ongoing treatment with tablets, which of the following are you using?
    □ Methotrexate (methotrexate)
    □ Sandimmun (cyclosporine)
    □ Imurel (azathioprine)
    □ Neotigason (acitretin)
    □ Toctino (alitretinoin)
    □ CellCept (mycophenolate mofetil)
    □ Olumiant (baricitinib)
    □ Cibinqo (abrocitinib)
    □ RINVOQ (upadacitinib)
    □ I don’t use tablets

18. If you have ongoing treatment with injections, which of the following are you using?
    □ Dupixent (dupilumab)
    □ Xolair (omalizumab)
    □ I don’t use injections

19. Do you have other ongoing treatments for atopic eczema?
    ○ I don’t use other treatments
    ○ Yes, and by other treatments I mean… → FREE-TEXT ANSWER

In the following, you will find questions about topical steroid withdrawal (TSW).

20. Are you familiar with the term topical steroid withdrawal (TSW)?
    ○ Yes
    ○ No → QUESTIONNAIRE TERMINATED

21. How would you define the term TSW? (maximum two lines of text) → FREE-TEXT ANSWER

22. Are you currently experiencing skin symptoms which you believe are caused by TSW?
    ○ Yes
    ○ No → FORWARDED TO ITEM 24

23. If your answer to the previous question was ‘No’, have you previously experienced skin symptom which you believed were caused by TSW?
○ Yes, within the last 3 months
○ Yes, 3–6 months ago
○ Yes, 6–12 months ago
○ Yes, more than 12 months ago
○ No, never → QUESTIONNAIRE TERMINATED

24. How many episodes of TSW have you experienced to date? (An episode is considered resolved after two months without symptoms)
○ One episode
○ Two episodes
○ Three episodes
○ Four episodes
○ Five or more episodes
○ No episode → QUESTIONNAIRE TERMINATED

25. What signs and symptoms from your skin do you associate with TSW? Please base your answer on your most recent TSW episode.
□ Redness
□ Bumps
□ Pustules
□ Dryness
□ Peeling skin
□ Oozing
□ Crusting
□ Itch
□ Burning
□ Stinging
□ Pain
□ Numbness
□ Increased sensitivity
□ Sleep disturbance
□ Other (please specify) → FREE-TEXT ANSWER

26. Which of the following skin areas are affected by TSW?

□ Face

□ Scalp

□ Ears

□ Neck

□ Chest

□ Armpits

□ Abdomen

□ Back

□ Upper arms

□ Forearms

□ Hands

□ Buttocks

□ Genital area

□ Groin

□ Thighs

□ Lower legs

□ Feet

27. Who was the first person to confirm that you were suffering from TSW?

○ General practitioner

○ Dermatologist

○ Practitioner of complementary medicine (used in addition to prescribed treatment) and/or alternative medicine (used instead of prescribed treatment), for instance natural remedies, supplements, diets, acupuncture, homeopathy or other

○ Representative from patient advocacy group

○ Social media contact

○ Family member or relative

○ Friend/friends

○ I myself

○ Other specialist medical practitioner (please specify) → FREE-TEXT ANSWER

28. Have you previously consulted a healthcare provider for TSW?

○Yes

○No → FORWARDED TO ITEM 34

Which healthcare provider/s have you consulted for TSW?

29. General practitioner
    ○ Previous contact, not ongoing
    ○ Ongoing contact
    ○ Never had contact

30. Dermatologist
    ○ Previous contact, not ongoing
    ○ Ongoing contact
    ○ Never had contact

31. Other specialist practitioner
    □ Previous contact, not ongoing
    □ Ongoing contact
    □ Never had contact
    □ If you have consulted with another specialist practitioner/s, please specify! → FREE-TEXT ANSWER

32. Practitioner of complementary medicine (used in addition to prescribed treatment) and/or alternative medicine (used instead of prescribed treatment), for instance natural remedies, supplements, diets, acupuncture, homeopathy or other
    ○ Previous contact, not ongoing
    ○ Ongoing contact
    ○ Never had contact

33. What investigations have been performed by your healthcare provider in regard to your TSW?
    □ Medical history on previous diseases and treatments
    □ Physical examination of the affected skin areas by a doctor
    □ Physical examination of all skin areas by a doctor
    □ Skin biopsy
    □ Blood tests
    □ Contact allergy test (applied on your back and checked after a few days)
    □ No investigations have been performed
    □ Other actions (please specify) → FREE-TEXT ANSWER

34. If you haven't consulted a healthcare provider for TSW, what is the reason for that?
    □ It hasn't occurred to me that a healthcare provider could help me
    □ I am afraid that the medical staff has insufficient knowledge of TSW

□ I am afraid that the medical staff does not believe that TSW exists

□ I have been advised not to consult a healthcare provider

□ I don’t believe that healthcare providers can offer the right treatment for TSW

□ Other (please specify) → FREE-TEXT ANSWER

35. Which sources have you utilised for information about TSW?

□ TSW-themed web pages

□ Facebook groups

□ Instagram

□ Twitter

□ Books/magazines/journals

□ TV/radio

□ Podcasts

□ Other social media/other sources (please specify) → FREE-TEXT ANSWER

36. What do you believe to be the trigger factor for **your** TSW symptoms?

□ Topical cortisone (ointment/cream/lotion/pharmaceutical solutions

□ Cortisone tablets

□ Other treatment or cause (please specify) → FREE-TEXT ANSWER

If a medical treatment triggered your TSW symptoms, how long had you been using that treatment continuously before symptoms arose? (Continuously means without any interruption of four weeks or more.)

37. Topical cortisone

○ Less than 3 months

○ 3–6 months

○ 7–12 months

○ More than 12 months

○ I don’t believe that topical cortisone triggered my TSW symptoms

38. Cortisone tablets

○ Less than 3 months

○ 3–6 months

○ 7–12 months

○ More than 12 months

○ I don’t believe that cortisone tablets triggered my TSW symptoms

39. Other treatment
    - ○ Less than 3 months
    - ○ 3–6 months
    - ○ 7–12 months
    - ○ More than 12 months
    - ○ I haven’t indicated other treatment

In the following, you will find statements about how TSW affects your life. Please rate how applicable each statement is to your situation on a scale from 0 (does not describe my situation at all) to 10 (describes my situation perfectly).

40. I avoid social contact due to TSW symptoms
    → MARK ON VISUAL ANALOGUE SCALE 0–10

41. TSW symptoms disturb my sleep
    → MARK ON VISUAL ANALOGUE SCALE 0–10

42. I have difficulty concentrating during the day due to TSW symptoms
    → MARK ON VISUAL ANALOGUE SCALE 0–10

43. I have absence from school or work due to TSW symptoms
    → MARK ON VISUAL ANALOGUE SCALE 0–10

44. I avoid intimacy with a partner due to TSW symptoms
    → MARK ON VISUAL ANALOGUE SCALE 0–10

45. TSW symptoms cause me anxiety
    → MARK ON VISUAL ANALOGUE SCALE 0–10

46. TSW symptoms make me feel depressed
    → MARK ON VISUAL ANALOGUE SCALE 0–10

47. What kind of help and support would you like from the healthcare system?
    → FREE-TEXT ANSWER

Thank you for your participation. If you know anyone else affected, please feel free to send them the link to the questionnaire.
