# Supplementary material for: Health Care Utilization in Patients With Atopic Dermatitis Experiencing Topical Steroid Withdrawal: Observational Cross-Sectional Social Media Questionnaire Study
Source: JMIR Form Res. 2025 Dec 31;9:e85183. doi: 10.2196/85183 (PMC12755344; doi:10.2196/85183)

Multimedia Appendix 2. Flowchart for questionnaire items and flowchart for questionnaire participation.

Figure S1. Flowchart for questionnaire items.

STOP represents automatic termination of the questionnaire when the chosen item response does not meet the inclusion criteria: previous or ongoing atopic dermatitis and previous or ongoing topical steroid withdrawal. A yellow box represents an item where the chosen response determines the subsequent item.

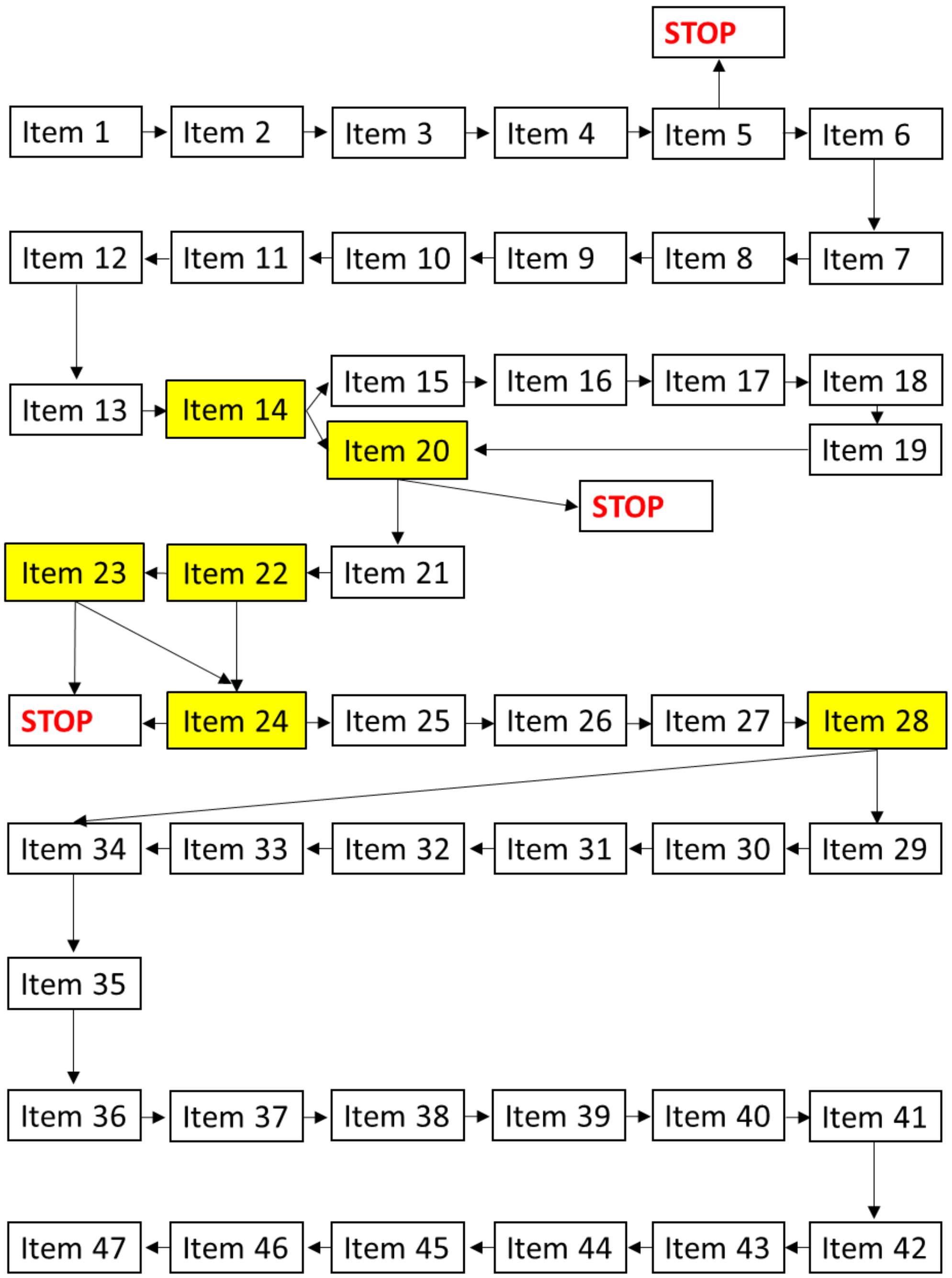

Figure S2. Flowchart for questionnaire participation.

The questionnaire was automatically terminated if a participant did not indicate previous/ongoing atopic dermatitis or previous/ongoing topical steroid withdrawal. There were no or very few answers to topical steroid withdrawal-related items in the questionnaires classified as incomplete.

AD: atopic dermatitis; TSW: topical steroid withdrawal

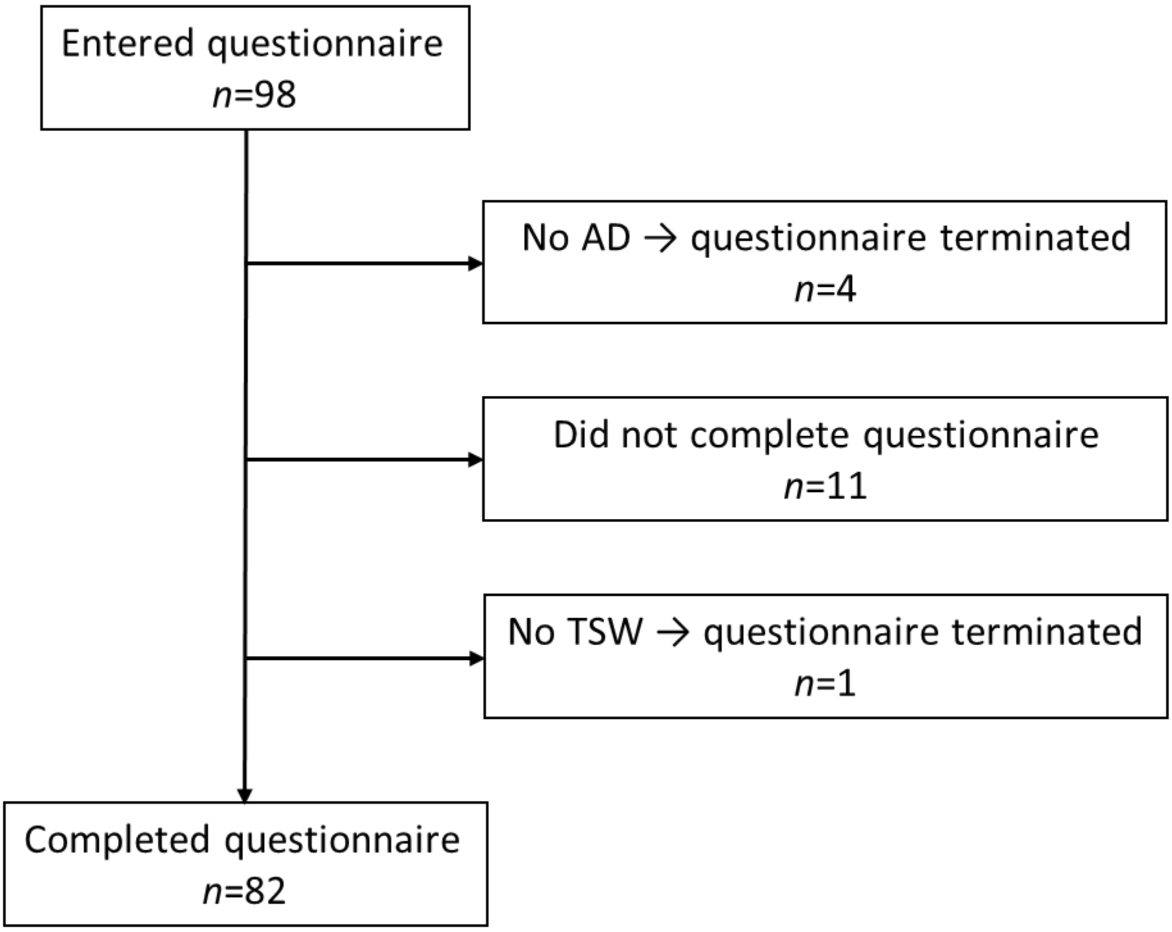

Supplement: Multimedia Appendix 2 [file formative-v9-e85183-s002.pdf]
