# Supplementary material for: Health Care Utilization in Patients With Atopic Dermatitis Experiencing Topical Steroid Withdrawal: Observational Cross-Sectional Social Media Questionnaire Study
Source: JMIR Form Res. 2025 Dec 31;9:e85183. doi: 10.2196/85183 (PMC12755344; doi:10.2196/85183)
Supplement: Checklist 1 [file formative-v9-e85183-s003.docx]

Checklist 1. Checklist for Reporting Results of Internet E-Surveys (CHERRIES).

| 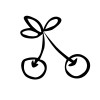 | **Checklist for Reporting Results of Internet E-Surveys (CHERRIES)** | |
| --- | --- | --- |
| ***Item Category*** | ***Checklist Item*** | ***Explanation*** |
| **Design** |  |  |
|  | Describe survey design | Observational, cross-sectional, social media questionnaire survey.  Inclusion criteria: Individuals aged ≥18 years with atopic dermatitis and prior or current experience of symptoms they attribute to topical steroid withdrawal (TSW).  The sample is a convenience sample. |
| **IRB (Institutional Review Board) approval and informed consent process** |  |  |
|  | IRB approval | Ethical approval for the study, including the study protocol, was sought with a request for an advisory statement from the Swedish Ethical Review Authority. As the study did not involve any intervention or processing of identifiable personal data, it was not subject to formal ethical review under Swedish legislation. The advisory statement from the Swedish Ethical Review Authority confirmed that there were no ethical objections to the study (application number Dnr 2023-00189-01). |
|  | Informed consent | Informed consent described the purpose of the study, which data were stored, how data were stored, how the data would be used, and that the participants were anonymous to the investigators. |
|  | Data protection | A description of the management of data, summarized below, was included in the informed consent.  Answers were given anonymously and were the only data collected in the study. The answers could not be traced back to the participant by the research group or anyone else. Data were saved on a password-protected computer behind a firewall and were only accessible to those researchers who analysed and presented the data.  The informed consent also described the right to file a complaint with the supervisory authority, IMY, the Swedish Authority for Privacy Protection, should a participant be unsatisfied with the way that data were processed.  The informed consent is found in Multimedia Appendix 2. |
| **Development and pre-testing** |  |  |
|  | Development and testing | A 47-item questionnaire was constructed and tested in Swedish in SurveyMonkey® (SurveyMonkey Inc, San Mateo, California, USA, [www.surveymonkey.com](http://www.surveymonkey.com)). In short, the questionnaire was designed and modified in a stepwise fashion where items were consecutively evaluated by the authors, a focus group of dermatologists from the steering group of SwedAD (the Swedish nationwide registry for patients with AD receiving systemic pharmacotherapy), and a focus group of patients with atopic dermatitis recruited from the Swedish Asthma and Allergy Association. Questionnaire items were multiple-choice or open-ended. The usability and technical functionality of the electronic questionnaire was tested before fielding. |
| **Recruitment process and description of the sample having access to the questionnaire** |  |  |
|  | Open survey versus closed survey | Open survey. |
|  | Contact mode | The initial contact with the potential participants was made through a post explaining the purpose of the study in a Swedish, private, TSW-themed Facebook group. The post included a link to the survey. The post and the link could be shared freely through social media by anyone with access to the Facebook group. |
|  | Advertising the survey | The Facebook group chosen for the fielding of the questionnaire was the largest Swedish online community for TSW known to the authors at the time.  The administrator of the Facebook group was contacted by the patient representative in the research group with a request to post a study announcement and a link to the questionnaire. The post was made on 24 April 2023, describing the aim of the study and encouraging dissemination of the link through sharing on social media where relevant. Reposts were made on 9 May 2023 and 17 May 2023 as reminders. The questionnaire remained open for four consecutive weeks.  The questionnaire items are found in Multimedia Appendix 2. |
| **Survey administration** |  |  |
|  | Web/e-mail | The survey was posted as a link on Facebook and could be shared on social media for four weeks. |
|  | Context | The survey was posted in a Swedish, private, TSW-themed Facebook group. Presumed members of the group were individuals with symptoms attributed to TSW, family and friends of those individuals, and individuals with an interest in this topic. Presumed reasons for joining the group were support and information.  Individuals with TSW are hard-to-define as there are no acknowledged criteria for the condition. They are also a hard-to-reach group as their presence in the healthcare system is unknown.  The aim of the study was to describe and and define different aspects of TSW as they are perceived by those who identify with the condition.  We sought as many individuals as possible with atopic dermatitis together with experiences of TSW.  This mode of recruitment can introduce bias associated with social media habits, severity of symptoms, and engagement in advocacy groups. |
|  | Mandatory/voluntary | Voluntary survey. |
|  | Incentives | No incentives were offered. |
|  | Time/Date | The survey was open from 24 April to 21 May 2023. |
|  | Randomization of items or questionnaires | Items were not randomized or alternated. |
|  | Adaptive questioning | Adaptive questioning was used. Figure S1 in Multimedia Appendix 3 A presents the flowchart for questionnaire items. |
|  | Number of Items | The questionnaire contained 47 items. |
|  | Number of screens (pages) | 5-6 items were screened on each page. |
|  | Completeness check | Data from all questionnaires entered and answered, incompletely or completely, were submitted (n=98) and reviewed. Only completed questionnaires (n=82) were included in the final analysis.  The incomplete questionnaires (n=16) were distributed as follows:  Atopic dermatitis criteria not fulfilled (n=4)  TSW criteria not fulfilled (n=1)  Completeness rate very low with no or very few answers to TSW-related items (n=11) |
|  | Review step | Participants could go back, review, and revise the items screened on one page (5-6 items). It was not possible to go back to previous pages. |
| **Response rates** |  |  |
|  | Unique site visitor | Participants were assigned unique respondent identification numbers. An electronic device could only be used once to submit an entered questionnaire. |
|  | View rate (Ratio of unique survey visitors/unique site visitors) | The number of views of the posts with the study announcement were not recorded. The dissemination of the study announcement through sharing within or outside the Facebook group is unknown to the authors.  The data from all entered questionnaires, even if incompletely filled out, were submitted. |
|  | Participation rate (Ratio of unique visitors who agreed to participate/unique first survey page visitors) | Unknown. |
|  | Completion rate (Ratio of users who finished the survey/users who agreed to participate) | Data from all questionnaires entered and answered, incompletely or completely, were submitted (n=98). The questionnaire was automatically terminated if a respondent did not fulfil the inclusion criteria.  Completed questionnaires (n=82) were included in the analyses.  The completion rate was 84 %.  Figure S2 in Multimedia Appendix 3 presents the flowchart for the completion rate. |
| **Preventing multiple entries from the same individual** |  |  |
|  | Cookies used | Cookies were not used. |
|  | IP check | An electronic device (one IP address) could only be used once to submit an entered questionnaire. |
|  | Log file analysis | No other techniques to analyse the log file for identification of multiple entries were used. |
|  | Registration | Open survey, non-applicable. |
| **Analysis** |  |  |
|  | Handling of incomplete questionnaires | Incomplete questionnaires (n=16) were reviewed:  Atopic dermatitis criteria not fulfilled (n=4)  TSW criteria not fulfilled (n=1)  Completeness rate very low with no or very few answers to TSW-related items (n=11)  Only completed questionnaires were included in the final analysis. |
|  | Questionnaires submitted with an atypical timestamp | Timestamps were not recorded. |
|  | Statistical correction | The results are presented with descriptive statistics. |
